# Assessing Quality of Referrals to a Community-Based Chronic Pain Clinic

**DOI:** 10.1080/24740527.2024.2402700

**Published:** 2024-10-28

**Authors:** Angela Mailis, Amna Rafiq, Amol Deshpande, S. Fatima Lakha

**Affiliations:** a Division of Physical Medicine & Rehabilitation, Department of Medicine, University of Toronto, Toronto, Ontario, Canada; b Pain and Wellness Centre, Vaughan, Ontario, Canada; c Health Sciences, Queens University, Kingston, Ontario, Canada; d Quality and Innovation, Department of Family and Community Medicine, University of Toronto, Toronto, Ontario, Canada; e Institute of Medical Sciences, University of Toronto, Toronto, Ontario, Canada

**Keywords:** rejected referrals, chronic pain, primary care providers (PCPs)

## Abstract

**Introduction:**

Because patients with chronic pain are complex, with significant medical and psychiatric comorbidities, referrals to specialty pain clinics are often necessary. The present study explores the quality of information submitted and the profile of referring physicians associated with rejected patient referrals by a community pain clinic.

**Methods:**

A retrospective cross-sectional study was conducted on a series of consecutive new patient referrals rejected by a noninterventional community pain clinic (November 2021–June 2022). Data were collected on the reasons for rejected referrals and physicians responsible for these referrals using the public database of the College of Physicians and Surgeons of Ontario.

**Results:**

During the study period, 120 new referrals made by 99 physicians (88% primary care providers, or PCPs; male : female ratio 1:1.2; 53% Canadian university graduates) were rejected because of inadequate information (62%) or because they were inappropriate (38%). Only 46% of the rejected referrals were resubmitted within a median of 7 days (range 0–96 days) and accepted. Half of the non-resubmitted referrals could have been accepted if the referring provider had sent in the missing information.

**Conclusion:**

A significant number of referrals to our pain clinic (primarily from PCPs) are rejected for mainly avoidable reasons. The process of rejected referrals and resubmissions requires 92 to 126 h of additional staff time/year. Without additional health care resources, our study highlights simple but effective improvements in the referral process that could facilitate patient care, avoid unnecessary delays, and decrease possible sources of patient complaints.

## Résumé

**Introduction**: Étant donné la complexité des patients atteints de douleur chronique, qui requièrent une prise en charge médicale adaptée et présentent souvent des comorbidités psychiatriques, il est souvent nécessaire de les référer à des cliniques spécialisées en matière de prise en charge de la douleur. Cette étude examine la qualité des informations fournies et le profil des médecins référents associés aux patients référés qui ont été refusés par une clinique communautaire de la douleur.

**Méthodes:** Une étude transversale rétrospective a été menée sur une série de nouvelles références de patients refusées par une clinique communautaire non interventionnelle de la douleur (novembre 2021– juin 2022). Des données ont été collectées sur les motifs de refus de ces références ainsi que sur les médecins responsables de ces références, en utilisant la base de données publique du Collège des médecins et chirurgiens de l'Ontario.

**Résultats:** Au cours de la période à l’étude, 120 nouvelles références effectuées par 99 médecins (88 % étaient des fournisseurs de soins primaires; le ratio hommes-femmes était de 1:1,2; 53 % étaient des diplômés universitaires canadiens) ont été refusées en raison d'informations insuffisantes (62 %) ou parce qu'elles étaient inappropriées (38 %). Seulement 46 % des références refusées ont été soumises à nouveau dans un délai médian de sept jours (intervalle de 0 à 96 jours) et acceptées. La moitié des références qui n’ont pas été soumises à nouveau auraient pu être acceptées si le fournisseur référent avait envoyé les informations manquantes.

**Conclusion:** De nombreuses références acheminées à notre clinique de la douleur (principalement par des fournisseurs de soins primaires) sont refusées pour des raisons le plus souvent évitables. Le processus de référence et de nouvelle soumission des références refusées nécessite de 92 à 126 heures de temps de personnel supplémentaire/an. Notre étude met en évidence des améliorations simples mais efficaces dans le processus de référencement qui pourraient faciliter les soins aux patients, éviter des retards inutiles et réduire les sources possibles de plaintes des patients, sans nécessiter de ressources supplémentaires en soins de santé.

## Introduction

In many countries, referral of patients from general physicians to specialists is necessary to access and control health resources. Accurate and detailed communication between all physicians is critical to ensure efficient and high quality of care delivery, no matter how simple or complex the patient or specialist’s involvement. Referral processes vary significantly, not only across specialties but among specialists within a particular clinical domain and even within a geographic region.^1^ There are no best practices outlining the most effective method of communication or content required for a given situation.

In Canada, physicians refer patients for specialty care by selecting the most appropriate specialists or clinic to address the patient’s condition and fax the relevant information.^2^ Specialists schedule appointments after assessing the patient’s files and prioritizing them by medical urgency.^3^ Specialists may reject referrals for several reasons including but not limited to insufficient information,^4^ protracted wait lists, or referrals made outside the scope of practice etc.

Various data sources, such as chart audits, questionnaires, health administration databases, and electronic health records, have been used to study referral trends from primary care providers (PCPs) to specialists in many countries.^5–10^ Specifically, in Canada, several studies have examined referral patterns.^7,11,12^ Though the most frequent PCP referrals appear to be sent (in order of rank) to gastroenterology, obstetrics and gynecology, dermatology, and general surgery,^8^ little is known about referral practices between PCPs and pain clinics in Canada. In a large survey of American physicians, only 34.8% of consultants stated they received referrals from primary care containing relevant and adequate clinical details.^13^

Because patients with chronic pain are complex with significant medical and mental health challenges and associated high levels of health care utilization, referrals to specialty pain clinics are necessary in many cases.^14^ Previous evidence has documented prolonged wait times for patients with chronic pain in Canada, with calls for more resources to alleviate this issue.^15^ Referrals lacking adequate information or inappropriate for the scope and expertise of a pain clinic may cause unnecessary delays or initial rejection of referred patients and duplicative administrative load and generate frustration for patients, specialists, and clinic staff.^16^ An effective and efficient referral process could help to alleviate the personal, emotional, and any economic burden related to unnecessary wait times.

This study aims to identify the reasons behind the rejection of patient referrals by a community pain clinic and describe the profile of physicians who submitted referrals that were rejected.

## Methods

### Design

A retrospective cross-sectional study was conducted on a series of consecutive new patients with chronic pain referred to and initially rejected by a community-based pain clinic between November 2021 and June 2022. The study was approved by the University of Toronto Research Ethics Board (REB No. 43224). All patients signed informed written consent outlining the anonymous use of their data in an aggregate format for research purposes.

### Setting and referral process

The Pain and Wellness Center (PWC) was founded in 2014 as a community-based pain clinic in the province of Ontario, Canada, serving adults of all ages and youth (from age 12 to 18). It provides pain consultations, investigations, treatments, and multi/interdisciplinary pain management but does not perform “nerve blocks”; for example, trigger point injections, spinal injections, or injections in peripheral nerves or roots (hence it is designated as a noninterventional pain clinic). The center receives referrals that range from common medical conditions such as sports injuries, herniated disc with sciatica, rotator cuff injuries or osteoarthritis to extremely complex cases with severe biomedical and/or psychiatric comorbidities,^17,18^ unclear diagnosis, functional neurological disorders, and widespread pain conditions with severe disability. The PWC has a specific referral form in PDF nonfillable format (Appendix 1), accessible on the clinic’s website. The form requests basic information on the patient and the referring physician, a succinct summary of the patient’s history (e.g., Cumulative Patient Profile or other summary), relevant imaging, and related consultations, if available. The form also clearly indicates services that are not accessible through the clinic (e.g., cannabis, interventional procedures such as nerve blocks, etc.), and patients who have been seen by several other clinics, investigated, and treated are not accepted to avoid duplication of health utilization (“nothing to offer”).

**Figure 1.**
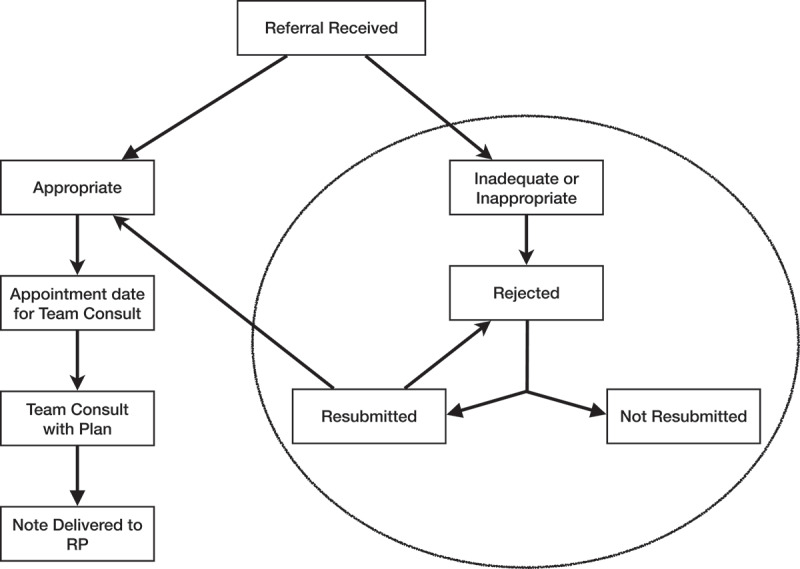
Referral process.

The diagram in Figure 1 illustrates the referral process. The study relates to the portion of the process encircled in the diagram. If a referral is rejected, a specific form indicating the reason(s) for rejection is faxed to the referring physician/clinic.

### Data collection

Data were collected on all PCPs and specialists (collectively called referring physicians, or RPs) who were responsible for the rejected referrals. A minimal patient data set (e.g., date of referral, patient name and gender) was collected through the clinic’s electronic medical record (EMR) system.

Reasons for rejection (outlined in our referral form) were cataloged using prespecified codes. A referral rejection form highlighting the reason(s) for inappropriate referral was sent to the RP for all such submissions (Appendix 2).

To provide context to the overall referral rejection rate in our clinic, we retrieved certain data from our quarterly submissions to the provincial Ministry of Health and Long-Term Care.

For physicians whose referrals were rejected, provider demographic information (i.e., sex, university associated with MD degree, specialty, language(s) spoken, and years elapsed between graduation and start of practice) was collected using the College of Physicians and Surgeons of Ontario (CPSO) public database. Time-to-practice was calculated as the time from the year of graduation to the year of independent practice. The latter was defined as (1) most recent date of license granted after specialty certification by the CPSO, including PCPs holding Certification by the College of Family Physicians (CCFP designation) or (2) earliest license registration with CPSO for those who had no specialty designation or (3) date of most recent independent practice in Ontario for foreign trained physicians.

The study also categorized rejected referrals by physicians who graduated from a Canadian medical school and those who graduated from a medical school outside Canada,^19^ the former classified as Canadian university (CU) graduates and the latter as foreign university (FU) graduates.

### Data analysis

All data were analyzed using SPSS v16.0.^20^ Descriptive statistics were used for physicians’ demographic characteristics and referral status. Categorical variables were summarized using proportions; continuous variables were reported using mean and standard deviation. Fisher’s exact tests or the Pearson χ^2^ test were employed to compare categorical variables. For continuous variables, a *t*-test was used. The ratio analysis made use of the χ^2^ goodness-of-fit test. The two-sided *P* value of 0.05 was used to determine minimal statistical significance at a 95% confidence range. When the denominator was different because of missing data, the exact number was shown in brackets. For referrals that were rejected and then resubmitted with additional information and accepted, we calculated time from initial rejection date to acceptance.

## Results

### Reasons for rejected referrals

The two most frequent reasons for rejected referrals were (1) inadequate information with little or no relevant content, accounting for 62% of rejected referrals (74/120), and (2) inappropriate referrals, accounting for 38% of rejected referrals (46/120; Table 1).

**Table 1.** Primary reasons for rejected referrals.

First rejection (*N* = 120 referrals)	*N*	%
#1 Inadequate	74	62
#2 Miscellaneous^a^	17	14
#3 Not a block clinic	8	7
#4 Nothing to offer	8	7
#5 Outside the scope^b^	6	5
#6 Gastrointestinal or pelvic pains	5	5
*N*	118	

Note that 118/120 referrals were rejected for the above-cited six reasons.

^a^Examples: illegible handwriting; referral of a frail elderly person post hospital discharge; a patient with acute sciatica 155 km away with no imaging or medication list, etc.

^b^Referrals included podiatry, referral for neuromuscular rehab in a patient with stroke, pediatric referral, etc.

Forty-six percent of the rejected referrals (*n* = 55 referrals) were resubmitted within a median of 7 days (range 0–96 days) and accepted. The remaining 54% of rejected referrals (*n* = 65/120) by 49 RPs were not resubmitted within a 6-month window after the cutoff time for rejected referral collection. Of note, 49% of those referrals (*n* = 32/65) had been deemed inadequate (i.e., if the necessary information were to be forwarded, these referrals would have likely been accepted).

### Characteristics of physicians responsible for rejected referrals

In total, 99 physicians were responsible for 120 rejected referrals, corresponding to 118 unique patients (2 referrals were submitted twice). The male : female ratio of referring physicians was 1:1.2 (43% males and 57% females). The majority of referrals (*n* = 87/99) were submitted by PCPs. Fifty-three percent of RPs (*n* = 53) were CU graduates (Table 2). No differences between CU and FU graduates were seen regarding demographic data.

**Table 2.** Physicians’ characteristics.

		Gender of physicians	
Variables	All population (N = 99), n (%)	Male (N = 53), n (%)	Female (N = 46) (%)
Physicians’ university of graduation			
Canadian university	53 (54)	28 (53)	25 (54)
Foreign university	46 (47)	25 (47)	21 (46)
Physician specialty			
Family practice (CCFP and general practice)	87 (88)	44 (83)	43 (93)
Graduation to practice (in years), mean ± SD	7 ± 6.5	6.6 ± 6	7 ± 7

In terms of the FU graduates, 46% had graduated from South Central Asian University medical schools, 35% from the Caribbean, 9% from East Asia, 7% from Africa, and 4% from European medical schools. In terms of practice data, almost all CU graduates (98%) had been practicing for at least 10 years, as compared to only 48% of FU graduates. No differences between CU and FU female and male graduates were observed in terms of demographic characteristics and practice data.

With respect to languages spoken, all RPs spoke English, with 35 of them speaking English only. The remaining 64 physicians spoke one or more languages (besides English), with more than 80% fluent in languages spoken in Asia.

Fourteen out of 99 RPs accounted for more than one rejected referral. These 14 RPs (*n* = 13 registered as PCPs) submitted 33 rejected referrals. Fifty-seven percent of these physicians had a time from graduation to practice range of 1 to 5 years (fairly recent graduates). More than half of these RPs (57%) had graduated from Canadian universities. The demographic characteristics of these physicians were no different from the subgroups reported earlier.

Overall, only 29 RPs used our online PWC form, submitting 31 referrals. Despite use of our form, these referrals were lacking adequate information or were inappropriate (e.g., referral for injections, podiatry, etc.). Only 16 RPs whose referrals on our form had been rejected for lack of information resubmitted appropriate referrals (one per RP) and were subsequently accepted. The remaining 15 rejected referrals on our form from 13 RPs were never resubmitted, including 4 that could have been accepted if the requested information had been forwarded to us. Additionally, 16 RPs used Oscar (an EMR vendor used by PCPs but not specialists), and 54 RPs submitted a covering fax or some form of letter.

## Discussion

Our study shows that 62% of referrals to our pain clinic (primarily originating from PCPs) are rejected for mainly avoidable reasons and more than half of these rejected referrals are never resubmitted. Though difficulties faced by PCPs in seeking specialty care have been the focus in the literature, our study is the first in Canada to outline potential remediable causes, which could improve the efficiency of access to specialty pain care. This study also highlights a reverse situation: difficulties facing specialty pain clinics regarding poor quality of incoming referrals.

It is worth noting that the number of complex consultations referrals to our clinic (patients with multiple and severe biomedical and/or psychiatric disorders) increased substantially, from 15% of the referral pool in 2016 to >25% at present, based on our latest quarterly data submitted to the Ontario Ministry of Health and Long-Term Care. The complexity of the referred cases underscores the fact that transmission of basic and appropriate information from the referring physicians to the pain specialty clinic is mandated to avoid unnecessary tests and health care utilization.

This study, through an internal audit of our clinic, estimated the time spent by physicians and administrative staff in our center to triage rejected referrals. Each referral takes about 20 to 30 min for review, completion of the rejection form, and faxing it to the referring physician’s office, and review/acceptance of resubmitted referrals takes another 15 min. Extrapolating the current 7-month study results (120 rejected referrals, 55 resubmitted referrals) to a 12-month period, the process of rejection/resubmission of referrals accounts for 92 to 126 h of staff labor per year. This does not include time spent with distraught patients calling our clinic staff as to why appointments were delayed or could not be granted.

PCPs have reported difficulty accessing specialists and a lack of timely responses for appointments.^21^ According to a 2017 study,^22^ even after 7 weeks of referrals being sent, 36% of referrals were not acknowledged. Voiced complaints from PCPs regarding access to specialty care have increased post COVID, including complaints relating to very long wait lists, long time to deliver notes, and assigning tasks and investigations to primary care providers instead of ordering them themselves (illustrated by an excerpt from Dr. A. Stewart’s Canadian Healthcare Network Op Ed, February 14, 2023: “Family medicine is dumping ground for others people’s work”).^23^

Despite the presence of an online referral form, only 29% of RPs used it in this study. The form stipulates the types of patients we can service (and exclusionary criteria) and the requirement for basic information and attachment of imaging reports. Surprisingly, despite the use of our referral form, several such referrals were rejected due to omission of basic necessary information. It is unclear whether the poor results reflect workflow challenges with the form or form structure.

A further reason contributing to inappropriate or inadequate referral information could be related to poor understanding as to what services each pain clinic might offer. Because many pain clinics in the community offer interventions (i.e., injections, commonly known as “nerve blocks”), we often receive requests for injections even when this exclusionary criterion is clearly stipulated on the referral form and website. However, injections are a common indication for initiating referral by PCPs, and the issue has been explored and reported in an older publication by our group.^24^

We have attempted to remediate the challenge of rejected referrals by (1) attaching our referral form when we reject a referral to encourage future use by RPs; (2) accepting referrals without the form if submitted with basic background information, such as Cumulative Patient Profile or similar data, appropriate imaging, and previous relevant consultations (if they exist); and (3) employing a community navigator who has visited numerous family practices in a 50-km range to educate clinic administrative staff regarding basic requirements and leaving printed material and referral forms behind. Though these initiatives have been met with very limited success in terms of reducing the rejection rate, it is possible that they are not aligned with the current workflow of PCPs and their staff. However, currently, we are transforming our nonfillable PDF referral form to fillable form on our website. We intend to follow up the rate of rejected referrals once we upload the fillable form in the hope that our rate of rejections will decrease.

Although there are multiple facets to an effective referral system, one mechanism to improve the efficiency of referrals is the implementation of an electronic referral system.^25^ Though there are multiple components in this type of strategy, a relatively quick and low-cost solution might be to make the use of standardized referral forms mandatory and ensure that they are completed in full (force field function online on our website). This could reduce the number of rejections due to missing information and ensure that referrals are appropriate for the clinic’s scope of service.

Increasing accessibility of the form to providers by embedding a fillable PDF form in commonly used EMR systems for rapid and convenient use is a second possible solution that may increase adoption of this tool.

A potential (more difficult to apply) intervention to improve appropriate and adequate referrals to specialized pain facilities could be a province-wide ongoing education campaign for referring physicians and their administrative staff about available clinics and associated services. However, previous studies on referral guidelines for primary care to improve suitable referrals have been met with disappointing results.^26^ Moreover, such a service and, in particular, online directory of clinics (that requires regular maintenance) is a sizable enterprise in terms of labor and funding and rather difficult to materialize.

## Conclusions

The number of rejected referrals and reasons for rejections to a specialty pain clinic are problematic.

Overall, this study highlights the impact of inefficient or neglected processes that produce downstream challenges in timely patient care. Such rejected referrals create a significant and unnecessary amount of administrative burden for pain physicians and their administrative staff and the referring clinic or practice. Most important, the burden on patients is considerable because there is delay in provision of care or possibly no care at all. Though referral systems are complex entities, our study highlights two simple but likely effective improvements in the referral process that could facilitate patient care, avoid unnecessary delays, strengthen the collaboration of pain clinicians and referring providers, and decrease possible sources of complaints on behalf of patients.

## Conflicts of Interest

There are no financial relationships that might lead to a conflict of interest. A. Mailis does not have any conflicts of interest regarding the publication of this article. A. Despande does not have any conflicts of interest regarding the publication of this article. A. Rafique does not have any conflicts of interest regarding the publication of this article. S.F. Lakha does not have any conflicts of interest regarding the publication of this article.

## Supplementary Material

Appendix 2 June 10.docx

Appendix 1 feb05 .docx

Revised Rejected Referral MS July 22 2024 Trackchanges version.docx

Appendix 1 PWC Patient Referral Form.pdf

Revised Rejected Referral MS AMG June 13 2024 Track changes.docx

## Data Availability

Data will not be available in a public repository for reasons of patient privacy and confidentiality issues.
