# Supplementary figures and images for: Assessing Quality of Referrals to a Community-Based Chronic Pain Clinic
Source: Can J Pain. 2024 Oct 28;8(1):2402700. doi: 10.1080/24740527.2024.2402700 (PMC11520530; doi:10.1080/24740527.2024.2402700)

**Appendix 2: REJECTED REFERRAL FORM**

**
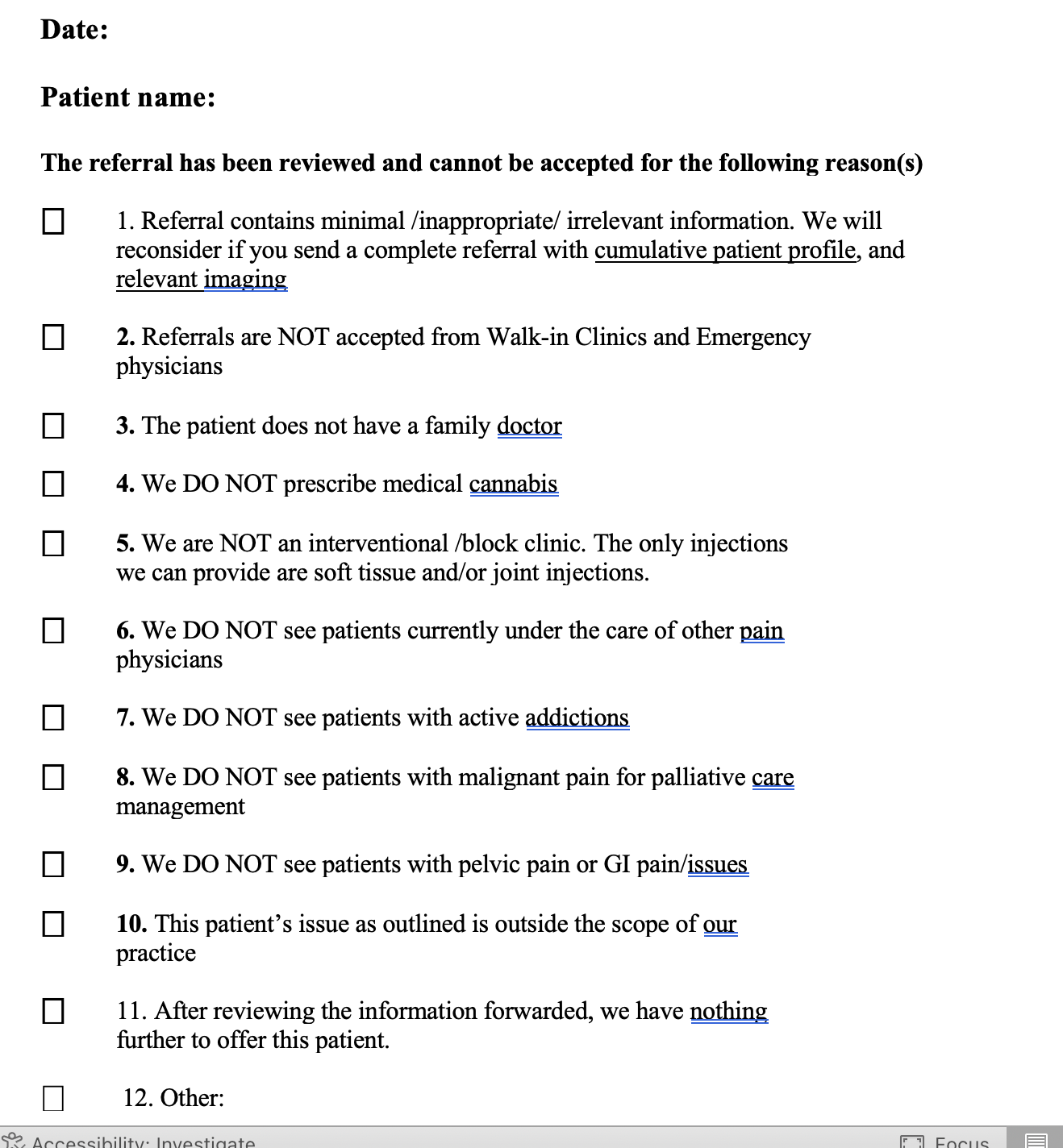
**

Supplement: Appendix 2 June 10.docx [file UCJP_A_2402700_SM3183.docx]

**Appendix 1**

**
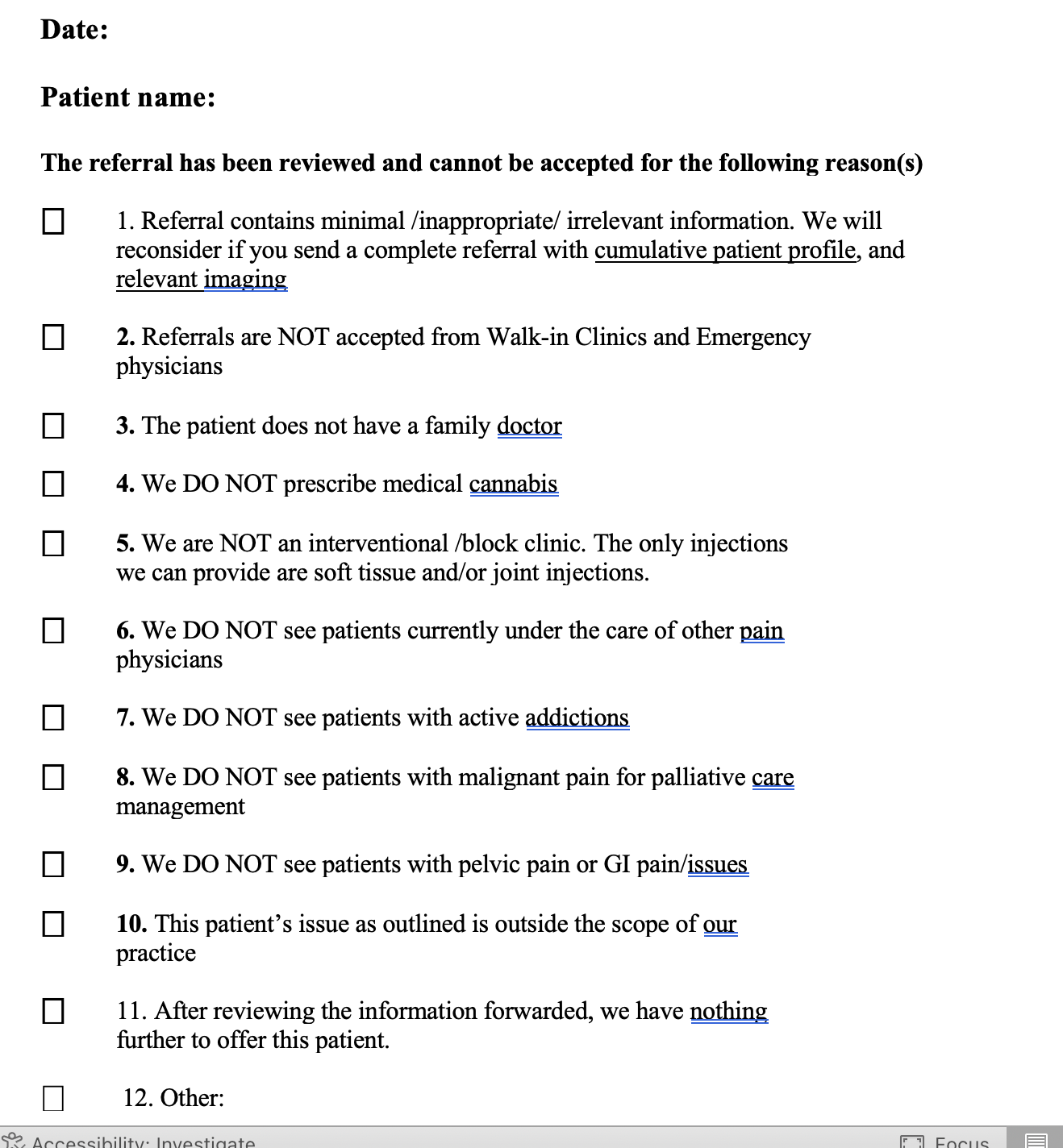
**

Supplement: Appendix 1 feb05 .docx [file UCJP_A_2402700_SM3182.docx]
