# Supplementary material for: Assessing Quality of Referrals to a Community-Based Chronic Pain Clinic
Source: Can J Pain. 2024 Oct 28;8(1):2402700. doi: 10.1080/24740527.2024.2402700 (PMC11520530; doi:10.1080/24740527.2024.2402700)
Supplement: Revised Rejected Referral MS July 22 2024 Trackchanges version.docx [file UCJP_A_2402700_SM3181.docx]

**Assessing Quality of Referrals to a Community-Based Chronic Pain Clinic**

**Angela Mailis, Amna Rafique, Amol Deshpande, S.Fatima Lakha**

1. Angela Mailis MD, MSc, FRCPC (PhysMed): Pain and Wellness Centre, Vaughan, Ontario, Canada; Clinical (Adjunct) Professor, Division of Physical Medicine & Rehabilitation, Department of Medicine, University of Toronto, Toronto Ontario Canada; 2301 Major Mackenzie Dr. West, Unit #101, Vaughan, Ontario, Canada

email: [angela.mailis@thepwc.ca](mailto:angela.mailis@thepwc.ca)

1. Amna Rafique BSc: Health Sciences, Queens University; Pain and Wellness Centre, Vaughan, Ontario, Canada;

email: 19ar51@queensu.ca

1. Amol Deshpande, MD, M.B.A: Assistant Professor, Quality and Innovation, Department of Family and Community Medicine, University of Toronto, Toronto, Ontario Canada

email: amol.deshpande@uhn.ca

1. Shehnaz Fatima Lakha MSc, Ph.D.**:** Pain and Wellness Centre, Vaughan, Ontario, Canada; Institute of Medical Sciences, University of Toronto, Toronto Ontario Canada; 2301 Major Mackenzie Dr. West, Unit #101, Vaughan Ontario Canada;

email: sfatima.lakha@utoronto.ca

Total #of pages: 15

Number of Tables: 3

References: 25

**Corresponding Author**

Angela Mailis MD, MSc, FRCPC (PhysMed)

Adjunct Clinical Professor, Dept. of Medicine,

University of Toronto

Director, Pain & Wellness Centre

The Pain & Wellness Centre

2301 Major Mackenzie Drive West Unit 101

Vaughan, ON L6A 3Z3

Phone: 1-800-597-5733

Email:angela.mailis@uhn.on.ca

**Introduction:** Since chronic pain patients are complex with significant medical and psychiatric comorbidities, referrals to specialty pain clinics are often necessary. The present study explores the quality of information submitted and the profile of referring physicians associated with rejected patient referrals by a community pain clinic.

**Methods:** A retrospective cross-sectional study was conducted on a series of consecutive new patient referrals, rejected by a non-interventional community pain clinic (November 2021– June 2022). Data were collected on the reasons for rejected referrals and physicians responsible for these referrals using the public database of CPSO.

**Results:** During the study period 120 new referrals made by 99 physicians (88% primary care providers or PCPs; male/female ratio 1:1.2; 53% Canadian University graduates) were rejected because of inadequate information (62%) or because they were inappropriate (38%). Only 46% of the rejected referrals were resubmitted within a median of 7 days (range 0-96 days) and accepted.

Half of the non-resubmitted referrals could have been accepted if the referring provider had sent in the missing information.

**Conclusion:** A significant number of referrals to our pain clinic (primarily from PCPs) are rejected for mainly avoidable reasons. The process of rejected referrals and resubmissions requires 92-126 hrs of additional staff time/year. Without additional healthcare resources, our study highlights simple but effective improvements in the referral process that could facilitate patient care, avoid unnecessary delays, and decrease possible sources of patient complaints.

Key words: Rejected referrals; chronic pain; Primary Care Providers (PCPs)

**INTRODUCTION**

In many countries, referral of patients from general physicians to specialists are necessary to access and control health resources. Accurate and detailed communication between all physicians is critical to ensure efficient and high quality of care delivery, no matter how simple or complex the patient or specialist’s involvement. Referral processes vary significantly, not only across specialties but among specialists within a particular clinical domain and even within a geographic region.^1^ There are no best practices outlining the most effective method of communication or content required for a given situation.

In Canada, physicians refer patients for specialty care by selecting the most appropriate specialists or clinic to address the patient’s condition and fax the relevant information.^2^ Specialists schedule appointments after assessing the patient’s files and prioritizing them by medical urgency.^3^ Specialists may reject referrals for several reasons including but not limited to insufficient information, ^4^ protracted wait lists, or referrals made outside the scope of practice etc.

Various data sources, such as chart audits, questionnaires, health administration databases, and electronic health records, have been used to study referral trends from primary care providers (PCPs) to specialists in many countries.^5,6,7,8,9,10^ Specifically, in Canada, several studies have examined referral patterns.^7,11,12^ While the most frequent PCP referrals appear to be sent (in order of rank) to gastroenterology, obstetrics and gynecology, dermatology, and general surgery,^8^ little is known about referral practices between PCPs and pain clinics in Canada. In a large survey of American physicians only 34.8% of consultants stated they received referrals from primary care containing relevant and adequate clinical details.^13^

Since chronic pain patients are complex with significant medical and mental health challenges and associated high levels of health care utilization, referrals to specialty pain clinics are necessary in many cases.^14^ Previous evidence has documented prolonged wait times for chronic pain patients in Canada with calls for more resources to alleviate this issue.^15^ Referrals lacking adequate information or inappropriate for the scope and expertise of a pain clinic may cause unnecessary delays or initial rejection of referred patients, duplicative administrative load, and generate frustration to the patients, specialists and clinic staff.^16^ An effective and efficient referral process could help to alleviate the personal, emotional, and any economic burden related to unnecessary wait times.

This study aims to identify the reasons behind the rejection of patient referrals by a community pain clinic and describe the profile of physicians who submitted referrals that were rejected.

**METHODS**

1. **Design**

A retrospective cross-sectional study was conducted on a series of consecutive new chronic pain patients referred to and initially rejected by a community-based pain clinic between November 2021 – June 2022. The study was approved by the University of Toronto, Research Ethics Board (REB number: 43224). All patients signed informed written consent outlining the anonymous use of their data in an aggregate format for research purposes.

1. **Setting and referral process**

The Pain and Wellness Centre (PWC) was founded in 2014, as a community-based pain clinic in the province of Ontario, Canada serving adults of all ages and youth (from age 12 to 18). It provides pain consultations, investigations, treatments, and multi/interdisciplinary pain management, but does not perform “nerve blocks”, e.g. trigger point injections, spinal injections or injections in peripheral nerves or roots (hence it is designated as non-interventional pain clinic). The centre receives referrals that range from common medical conditions such as sports injuries, herniated disc with sciatica, rotator cuff injuries or osteoarthritis; to extremely complex cases with severe biomedical and/or psychiatric comorbidities,^17, 18^ unclear diagnosis, Functional Neurological Disorders, and widespread pain conditions with severe disability. The PWC has a specific referral form in PDF non-fillable format (Appendix 1), accessible on the clinic’s website. The form requests basic information on the patient and the referring physician, a succinct summary of the patient’s history (e.g., Cumulative Patient Profile or other summary), relevant imaging, and related consultations, if available. The form also clearly indicates services that are not accessible through the clinic (e.g. cannabis, interventional procedures such as nerve blocks etc.), while patients who have been seen by several other clinics, investigated, and treated, are not accepted to avoid duplication of health utilization (“nothing to offer”).

The diagram below illustrates the referral process. The study relates to the portion of the process encircled in the diagram. If a referral is rejected, a specific form indicating the reason(s) for rejection is faxed to the referring physician/clinic. (Figure 1)


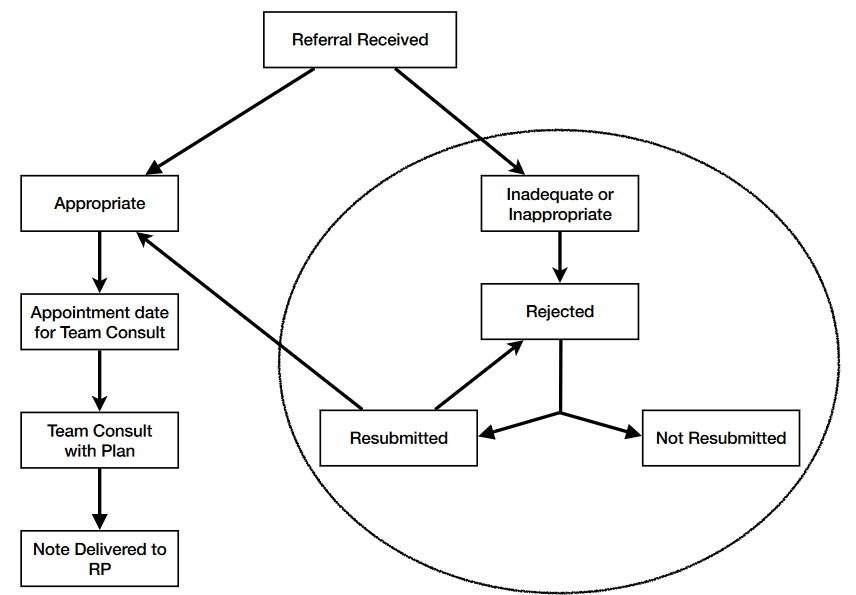


1. **Data Collection**

Data were collected on all primary care physicians (PCPs) and specialists (collectively called Referring Physicians = RPs) who were responsible for the rejected referrals. A minimal patient data set (e.g. date of referral, patient name and gender) was collected through the clinic’s electronic medical record (EMR) system.

Reasons for rejection (outlined in our referral form) were catalogued using pre-specified codes. A referral rejection form, highlighting the reason(s) for inappropriate referral was sent to the RP for all such submissions (Appendix 2).

To provide context to the overall referral rejection rate in our clinic, we retrieved certain data from our quarterly submissions to the provincial Ministry of Health and Long Term Care (MOHLTC).

For physicians whose referrals were rejected, provider demographic information (i.e., sex, university associated with MD degree, specialty, language(s) spoken, and years elapsed between graduation and start of practice) was collected using the College of Physicians and Surgeons of Ontario (CPSO) public database.  Time-to-practice was calculated as the time from the year of graduation to the year of independent practice. The latter was defined as: a) most recent date of license granted after specialty certification by the College of Physician and Surgeons of Ontario (CPSO), including PCPs holding Certification by the College of Family Physicians (CCFP designation); or b) earliest license registration with CPSO for those who had no specialty designation; or c) date of most recent independent practice in Ontario for foreign trained physicians.

The study also categorized rejected referrals by physicians who graduated from a Canadian medical schools and those who graduated from a medical school outside Canada,^19^ the former classified as *Canadian University (CU)* graduates and the latter as *Foreign University* *(FU)* graduates.

**Data Analysis**

All data were analyzed using SPSS (Statistical Package for the Social Sciences v.16.0, SPSS Inc., Chicago, IL, USA). Descriptive statistics were used for physicians' demographics and referral status. Categorical variables were summarized using proportions; continuous variables were reported using mean and standard deviation. Fisher's exact tests or the Pearson χ2 test were employed to compare categorical variables. For continuous variables, a t-test was used. The ratio analysis made use of the χ2 goodness-of-fit test. The two-sided P value of 0.05 was used to determine minimal statistical significance at a 95% confidence range. When the denominator was different because of missing data, the exact number was shown in brackets. For referrals that were rejected and then resubmitted with additional information, and accepted, we calculated time from initial rejection date to acceptance.

**RESULTS**

**Reasons for rejected referrals**

The two most frequent reasons for rejected referrals were: a) **Inadequate information** with little or no relevant content, accounting for 62% of rejected referrals (74/120), and b) **Inappropriate** **referrals** accounting for 38% (46/120) of rejected referrals (Table 1)**.**

Forty-six per cent of the rejected referrals (n=55 referrals) were resubmitted within a median of 7 days (range 0-96 days) and accepted. The remaining 54% of rejected referrals (n=65/120) by 49 RPs, were not resubmitted within a 6-month window after the cut-off time for rejected referral collection. Of note, 49% (n=32/65) of those referrals had been deemed inadequate (i.e., if the necessary information were to be forwarded, these referrals would have likely been accepted).

**Characteristics of physicians responsible for rejected referrals**

In total, 99 physicians were responsible for 120 rejected referrals, corresponding to 118 unique patients (two referrals were submitted twice). The male-female ratio of referring physicians was 1:1.2 (43% males and 57% females). The majority of referrals (n=87/99) were submitted by PCPs. Fifty-three percent of RPs (n=53) were CU graduates (Table 2). No differences between CU and FU graduates were seen regarding demographic data.

In terms of the FU graduates, 46% had graduated from South Central Asian University medical schools, 35% from the Caribbean, 9% from East Asia, 7% from Africa and 4% from European medical schools. In terms of practice data, almost all CU graduates (98%) had been practicing for at least 10 years as compared to only 48% of FU graduates. No differences between CU and FU female and male graduates were observed in terms of demographics and practice data.

With respect to languages spoken, all RPs spoke English with 35/99 of them speaking English only. The remaining 64 physicians spoke one or more languages (besides English), with more than 80% fluent in languages spoken in Asia.

Fourteen out of 99 RPs accounted for more than one rejected referral. These 14 RPs (n=13 registered as PCPs) submitted 33 rejected referrals. Fifty-seven per cent of these physicians had a time from graduation-to-practice range of 1-5 years (fairly recent graduates). More than half of these RPs (57%) had graduated from Canadian universities. The demographics of these physicians were no different from the subgroups reported earlier.

Overall, only 29/99 RPs used our online PWC form submitting 31 referrals. Despite use of our form, these referrals were lacking adequate information or were inappropriate (e.g. referral for injections, podiatry etc.). Only 16 RPs whose referrals on our form had been rejected for lack of information, resubmitted appropriate referrals (one per RP) and were subsequently accepted. The remainder 15 rejected referrals on our form from 13 RPs were never resubmitted including 4 that could have been accepted if the requested information had been forwarded to us. Additionally, 16/99 RPs used Oscar (an EMR vendor used by PCPs, but not specialists), while 54/99 RPs submitted a covering fax or some form of letter.

**DISCUSSION**

Our study shows that 62% of referrals to our pain clinic (primarily originating from PCPs) are rejected for mainly avoidable reasons and more than half of these rejected referrals are never re-submitted. While difficulties faced by PCPs in seeking specialty care have been the focus in the literature, our study is the first in Canada to outline potential remediable causes, which could improve the efficiency of access to specialty pain care. This study also highlights a reverse situation: difficulties facing specialty pain clinics regarding poor quality of incoming referrals.

It is worth noting that the number of complex consultations referrals to our clinic (patients with multiple and severe biomedical and/or psychiatric disorders) increased substantially, from 15% of the referral pool in 2016 to >25% at present, based on our latest quarterly data submitted to the Ontario Ministry of Health and Long Term Care. The complexity of the referred cases underscores even more the fact that transmission of basic and appropriate information from the referring physicians to the pain specialty clinic is mandated to avoid unnecessary tests and health care utilization.

This study, through an internal audit of our clinic, estimated the time spent by physicians and administrative staff in our centre to triage rejected referrals. Each referral takes about 20-30 min for review, completion of the rejection form, and faxing it to the referring physician’s office, while review/ acceptance of resubmitted referrals takes another 15 min. Extrapolating the current 7-month study results (120 rejected referrals, 55 resubmitted referrals) to a 12-month period, the process of rejection/ resubmission of referrals accounts for 92-126 hrs of staff labour per year. Additionally, this does not include time spent with distraught patients calling our clinic staff as to why appointments were delayed or could not be granted.

PCPs have reported difficulty accessing specialists and a lack of timely responses for appointments.^20^ According to a 2017 study, ^21^ even after seven weeks of referrals being sent, 36% of referrals were not acknowledged. Voiced complaints from PCPs regarding access to specialty care have increased post covid, including complaints relating to very long wait lists, long time to deliver notes, and assigning tasks and investigations to primary care providers instead of ordering them themselves (illustrated by an excerpt from Dr. A. Stewart’s Canadian Healthcare Network Op Ed, February 14, 2023: “Family medicine is dumping ground for others people’s work”).^22^

Despite the presence of an online referral form, only 29% of RPs used it in this study. The form stipulates types of patients we can service (and exclusionary criteria), and the requirement for basic information and attachment of imaging reports. Surprisingly, despite the use of our referral form, several such referrals were rejected due to omission of basic necessary information. It is unclear whether the poor results reflect workflow challenges with the form or form structure, respectively.

A further reason contributing to inappropriate or inadequate referral information could be related to poor understanding as to what services each pain clinic might offer. Since many pain clinics in the community offer interventions (i.e., injections, commonly known as “nerve blocks”), we often receive requests for injections even when this exclusionary criterion is clearly stipulated on the referral form and website. However, injections are a common indication for initiating referral by PCPs and the issue has been explored and reported in an older publication of our group.^23^

We have attempted to remediate the challenge of rejected referrals, by a) attaching our referral form when we reject a referral to encourage future use by RFs; b) accepting referrals without the form if submitted with basic background information, such as Cumulative Patient Profile or similar data, appropriate imaging and, previous relevant consultations (if they exist); and c) by additionally employing a Community Navigator who has visited numerous family practices in a 50 km range to educate clinic administrative staff regarding basic requirements and have even left printed material and referral forms behind. While these initiatives have been met with very limited success in terms of reducing the rejection rate, it is possible that they are not aligned with the current workflow of PCPs and their staff. However, currently, we are transforming our non-fillable PDF referral form to fillable on our website. We intend to follow up the rate of rejected referrals once we upload the fillable form in the hope our rate of rejections is decreased.

Although there are multiple facets to an effective referral system, one mechanism to improve the efficiency of referrals is the implementation of an electronic referral system.^24^ While there are multiple components in this type of strategy, a relatively quick and low-cost solution might be to make the use of standardized referral forms mandatory and ensure they are completed in full (force field function online on our website). This could reduce the number of rejections due to missing information and ensure that referrals are appropriate for the clinic’s scope of service.

In addition, increasing accessibility of the form to providers by embedding a fillable PDF form in commonly used Electronic Medical Record systems (EMRs) for rapid and convenient use, is a second possible solution that may increase adoption of this tool.

A potential (more difficult to apply) intervention to improve appropriate and adequate referrals to specialized pain facilities could be a province-wide ongoing education campaign for referring physicians and their administrative staff about available clinics and associated services. However, previous studies on referral guidelines for primary care to improve suitable referrals have been met with disappointing results.^25^ Moreover, such a service and in particular online directory of clinics (that requires regular maintenance) is a sizeable enterprise in terms of labour and funding and rather difficult to materialize.

**CONCLUSIONS**

The number of rejected referrals and reasons for rejections to a specialty pain clinic are problematic.

Overall, this study highlights the impact of inefficient or neglected processes that produce downstream challenges in timely patient care. Such rejected referrals create a significant and unnecessary amount of administrative burden for pain physicians and their administrative staff and the referring clinic or practice. Most importantly, the burden on patients is considerable as there is delay in provision of care or possibly no care at all. While referral systems are complex entities, our study highlights two simple but likely effective improvements in the referral process that could facilitate patient care, avoid unnecessary delays, strengthen the collaboration of pain clinicians and referring providers, and decrease possible sources of complaints on behalf of patients.

**DECLARATIONS:**

**Conflicts of Interest**

There are no financial relationships that might lead to a conflict of interest. Author A. Mailis does not have any conflicts of interest regarding the publication of this paper. Author A. Despande does not have any conflicts of interest regarding the publication of this paper. Author A. Rafique does not have any conflicts of interest regarding the publication of this paper. Author S.F. Lakha does not have any conflicts of interest regarding the publication of this paper.

**Funding Statement:**

This research received no specific grant from any funding agency in the public, commercial or not-for profit sectors.

**Authorship:**

All named authors meet the International Committee of Medical Journal Editors (ICMJE) criteria for authorship for this article, take responsibility for the integrity of the work as a whole, and have given their approval for this version to be published.

**Authors Contribution:**

A.M, A.R., A. D., and S.F.L. contributed to the design and implementation of the research, to the analysis of the results and to the writing of the manuscript.

**Data Availability:**

Data will not be available in a public repository due to the patients’ privacy and confidentiality issue.

**Ethical approval**

The study was reviewed and approved by the University of Toronto Human Ethics Research Committee (Protocol# 43224). Ethics and consent statements included in the method section.

**REFERENCES**

1. Liddy C, Singh J, Kelly R, Dahrouge S, Taljaard M, Younger J. What is the impact of primary care model type on specialist referral rates? A cross-sectional study. BMC Fam Pract. 2014; 15(1):22.
2. Grossman JM, Cross DA, Boukus ER, Cohen GR. Transmitting and processing electronic prescriptions: experiences of physician practices and pharmacies. Journal of the American Medical Informatics Association. 2012 May 1; 19(3):353-9. <https://doi.org/10.1136/amiajnl-2011-000515>
3. Keely E, Liddy C. Transforming the specialist referral and consultation process in Canada. CMAJ. 2019 Apr 15 [cited 2023 Aug 9]; 191(15):E408–9. Available from: <http://www.cmaj.ca/lookup/doi/10.1503/cmaj.181550>
4. Tobin-Schnittger P, O’Doherty J, O’Connor R, O’Regan A. Improving quality of referral letters from primary to secondary care: a literature review and discussion paper. Prim Health Care Res Dev. 2018 May [cited 2023 Aug 9]; 19(03):211–22. Available from: <https://www.cambridge.org/core/product/identifier/S1463423617000755/type/journal_article>
5. Corkum M, Viola R, Veenema C, Kruszelnicki D, Shadd J. Prognosticating in palliative care: a survey of Canadian palliative care physicians. J Palliat Care. 2011 Jun [cited 2023 Aug 9];27(2):89–97. Available from: <http://journals.sagepub.com/doi/10.1177/082585971102700204>
6. Chan BT, Austin PC. Patient, physician, and community factors affecting referrals to specialists in Ontario, Canada: a population-based, multi-level modelling approach. Medical Care. 2003 Apr [cited 2023 Aug 9];41(4):500–11. Available from: <https://journals.lww.com/00005650-200304000-00006>
7. Liddy C, Blazkho V, Mill K. Challenges of self-management when living with multiple chronic conditions: systematic review of the qualitative literature. Can Fam Physician. 2014 Dec; 60(12):1123–33.
8. Liddy C, Arbab-Tafti S, Moroz I, Keely E. Primary care physician referral patterns in Ontario, Canada: a descriptive analysis of self-reported referral data. BMC Fam Pract. 2017 Dec [cited 2023 Aug 9];18(1):81. Available from: <http://bmcfampract.biomedcentral.com/articles/10.1186/s12875-017-0654-9>
9. Forrest CB. Comparison of specialty referral rates in the United Kingdom and the United States: retrospective cohort analysis. BMJ. 2002 Aug 17 [cited 2023 Aug 9];325(7360):370–1. Available from: <https://www.bmj.com/lookup/doi/10.1136/bmj.325.7360.370>
10. Ringberg U, Fleten N, Deraas TS, Hasvold T, Førde O. High referral rates to secondary care by general practitioners in Norway are associated with GPs’ gender and specialist qualifications in family medicine, a study of 4350 consultations. BMC Health Serv Res. 2013 Dec [cited 2023 Aug 9];13(1):147. Available from: <https://bmchealthservres.biomedcentral.com/articles/10.1186/1472-6963-13-147>
11. Thind A, Stewart M, Manuel D, Freeman T, Terry A, Chevendra V, et al. What are wait times to see a specialist? an analysis of 26,942 referrals in southwestern Ontario. Healthc Policy. 2012 Aug;8(1):80–91.
12. Jaakkimainen L, Glazier R, Barnsley J, Salkeld E, Lu H, Tu K. Waiting to see the specialist: patient and provider characteristics of wait times from primary to specialty care. BMC Fam Pract. 2014 Dec [cited 2023 Aug 9];15(1):16. Available from: <https://bmcfampract.biomedcentral.com/articles/10.1186/1471-2296-15-16>
13. O'Malley AS, Reschovsky JD. Referral and consultation communication between primary care and specialist physicians: finding common ground. Arch Intern Med. 2011 Jan 10;171(1):56-65. doi: 10.1001/archinternmed.2010.480. PMID: 21220662
14. Foley HE, Knight JC, Ploughman M, Asghari S, Audas R. Association of chronic pain with comorbidities and health care utilization: a retrospective cohort study using health administrative data. Pain. 2021 Nov 1;162(11):2737-49.
15. Ansell D, Crispo JAG, Simard B, et al. Interventions to reduce wait times for primary care appointments: a systematic review. BMC Health Serv Res. 2017; 17:295. <https://doi.org/10.1186/s12913-017-2219-y>
16. Greenwood‐Lee J, Jewett L, Woodhouse L, Marshall DA. A categorisation of problems and solutions to improve patient referrals from primary to specialty care. BMC Health Serv Res. 2018;18(1):1–16.
17. Penlington, C., Urbanek, M., & Barker, S. (2019). Psychological theo- ries of pain. *Primary Dental Journal, 7*(4), 24–29.
18. Turk, D. C., & Okifuji, A. (2002). Psychological factors in chronic pain: Evolution and revolution. *Journal of Consult- ing and Clinical Psychology, 70*(3), 678–690.
19. Tsugawa Y, Jena AB, Orav EJ, Jha AK. Quality of care delivered by general internists in US hospitals who graduated from foreign versus US medical schools: observational study. BMJ. 2017;356:j273. <http://dx.doi.org/10.1136/bmj.j273>
20. Thind A, Freeman T, Thorpe C, Burt A, Stewart M. Family physicians’ satisfaction with current practice: what is the role of their interactions with specialists? Healthc Policy. 2009 Feb;4(3):e145-158.
21. Neimanis I, Gaebel K, Dickson R, Levy R, Goebel C, Zizzo A, et al. Referral processes and wait times in primary care. Can Fam Physician. 2017 Aug;63(8):619–24.
22. Stewart A. Family medicine is dumping ground for other peoples’ work. Canadian Healthcare Network Op Ed, February 14, 2023 <https://stewartmedicine.com/blog/family-medicine-dumping-ground/> (Accessed Nov 2023)
23. Lakha SF, Yegneswaran B, Furlan JC, Legnini V, Nicholson K, Mailis-Gagnon A. Referring patients with chronic noncancer pain to pain clinics: survey of Ontario family physicians. Can Fam Physician. 2011 Mar; 57(3):e106-112.
24. Seyed-Nezhad M, Ahmadi B, Akbari-Sari A. Factors affecting the successful implementation of the referral system: A scoping review. J Family Med Prim Care. 2021 Dec;10(12):4364-4375. doi: 10.4103/jfmpc.jfmpc_514_21. PMID: 35280649; PMCID: PMC8884299.
25. Kennedy AM, Aziz A, Khalid S, Hurman D. Do GP referral guidelines really work? Audit of an electronic urgent referral system for suspected head and neck cancer. Eur Arch Otorhinolaryngol. 2012 May; 269(5):1509-12. doi: 10.1007/s00405-011-1788-3. PMID: 21984061.
