# Supplementary material for: Assessing Quality of Referrals to a Community-Based Chronic Pain Clinic
Source: Can J Pain. 2024 Oct 28;8(1):2402700. doi: 10.1080/24740527.2024.2402700 (PMC11520530; doi:10.1080/24740527.2024.2402700)
Supplement: Appendix 1 PWC Patient Referral Form.pdf [file UCJP_A_2402700_SM3180.pdf]

PAIN & WELLNESS CENTRE

2301 Major Mackenzie Drive West, Unit 101, Vaughan, ON L6A 3Z3
1 (800) 597-5733 (Main Office) 1 (844) 358-9308 (Toll Free Fax)
info@thepwc.ca
www.thepwc.ca

Chronic Pain Referral for Assessment

Please note patients referred early after pain onset, have much higher chance of improvement.

Also NOTE that:
- We **do not** prescribe medical cannabis though we may refer our patients to cannabis clinics if there is proper indication.
- We **do not** see patients with severe or untreated psychopathology.
- We **do not** take over your patients' opioid prescribing.
- We reserve the right to **decline** referrals of patients that we think we are unable to help.
- We are **not** an Interventional/block clinic. The only injections we can provide are soft tissue and knee/ shoulder injections.
- Manual/Exercise Therapy, Psychological Therapy, Mindfulness, Massage & Naturopathic services are not covered by OHIP, and are paid by **extended health or privately.**
- Please avoid sending hand-written notes as they are difficult to read.

ESSENTIAL INFORMATION: *Please fill all fields on both pages*

Patient Information:

Name of Patient:________________________ HCN: ____________________

DOB: (DD/MM/YYYY):__________________ Sex of Patient: ________________

Full Mailing Address: ____

Patient Phone Number(s): ____

Does Patient Speak English? ☐ Minimal ☐ Fair ☐ Good/Fluent

Is Patient Treated Currently By Another Pain Facility? ☐ Yes ☐ No

Is Patient Currently On Disability? ☐ Yes (WSIB, car insurance, STD/LTD, CPP, other) ☐ No

Referring Provider Information:

Name of Referring Provider:____

OHIP Provider Number: ____

Phone Number of Referring Provider: _________________________________

Fax Number of Referring Provider: ___________________________________

Mailing Address of Referring Provider:_________________________________

____

(Continued on page 2)

Updated: Wednesday, March 4, 2020

Provisional Diagnosis (check ALL that apply):

- ☐ Chronic Widespread Pain
- ☐ Low Back Pain
- ☐ Other MSK Problem (Specify in Comments)
- ☐ Neuropathic Pain (Specify in Comments)
- ☐ Headaches (Specify in Comments)
- ☐ OTHER (Specify in Comments)

Comments

Most Important Pain Problem (Specify):

Comments

Is Pain The Result Of Any Of The Following? (check ALL that apply)

- ☐ Car Accident
- ☐ Work Injury
- ☐ Slip & Fall
- ☐ Disease Process (Specify in Comments)
- ☐ OTHER (Specify in Comments)

Comments

Report Important Medications:

(Include: Medications, Dose Present & Past)

Comments

Must Include Reports: **NO MORE THAN 20 PAGES OF COPIES**

- ☐ Cumulative patient profile/ CPP or comprehensive medical AND in particular PSYCHIATRIC history.
- ☐ CONSULTATIONS (Relevant to pain problem.
- ☐ X-rays, CAT, MRI, EMG/NCT, Bone scan, Ultrasound SPECIFIC TO CHIEF COMPLAINT.

**If referral is accepted, the patient will be notified within 2 weeks with appointment **
Incomplete referrals missing requested information will be returned to your office

Updated: Wednesday, March 4, 2020
